# The Increase of ROS Caused by the Interference of DEHP with JNK/p38/p53 Pathway as the Reason for Hepatotoxicity

**DOI:** 10.3390/ijerph16030356

**Published:** 2019-01-27

**Authors:** Yuanyuan Huang, Chuancheng Wu, Youbin Ye, Jingwen Zeng, Jianlin Zhu, Yuchen Li, Wenxiang Wang, Wenchang Zhang, Yiqin Chen, Hongyuan Xie, Hongmei Zhang, Jin Liu

**Affiliations:** 1 Department of Preventive Medicine, Fujian Provincial Key Laboratory of Environment factors and Cancer, Key Laboratory of Environment and Health, School of Public Health, Fujian Medical University, Xueyuan Road No.1, Minhou County, Fuzhou 350108, China; huangyy917@163.com (Y.H.); ppdostombt@hotmail.com (C.W.); yyb121000@163.com (Y.Y.); eightyu8@163.com (J.Z.); zhujianlinxy@126.com (J.Z.); lyc2000zs@163.com (Y.L.); wenchang2008@126.com (W.Z.); 13313752014@163.com (H.X.); 18516672467@163.com (H.Z.); 2 Department of Health Inspection and Quarantine, School of Public Health, Fujian Medical University, Xueyan Road No.1, Minhou County, Fuzhou 350108, China; wangwenxiang@fjmu.edu.cn (W.W.); 13123184166@163.com (Y.C.)

**Keywords:** DEHP, ROS, JNK/p38MAPK/p53, DNA methylation

## Abstract

As the most commonly used plasticizer, Di-(2-ethylhexyl)-phthalate (DEHP) exists everywhere in the environment due to the widespread use of polyvinyl chloride (PVC) in human life, and it is also a recognized environmental pollutant. Studies have proved the hepatotoxicity of DEHP, however the mechanism has not been adequately explored, especially the role of the reactive oxygen species (ROS) in it. In the present study, 21 day-old ICR mice were administered DEHP with dose of 0, 125, 250, and 375 mg/kg/day for 28 days by intragastrical gavage. After contamination, histopathology displayed that liver tissue were damaged mildly with the effect of DEHP; a significant increase of the serum liver function index (including aspartate transaminase (AST) and alanine transaminase (ALT)) were observed. Additionally, the level of lipid peroxidation markedly rise, especially ROS and malondialdehyde (MDA), but the activation of superoxide dismutase (SOD) was obviously decreased in mice liver. In addition, DEHP promoted the phosphorylation of JNK and p38MAPK proteins in mice liver, as well as increased the expression of p53 protein and decreased the level of DNA methylation in the p53 gene promoter region. These results indicated that the hepatotoxicity of mice caused by DEHP may be through activating the JNK/p38MAPK/p53 signaling pathway and further promoting the generation of ROS to induce lipid peroxidation in liver, and the role of DNA methylation may be inevitable.

## 1. Introduction

Di(2-ethylhexyl) phthalate (DEHP), the most widely used representatives in phthalic acid esters (PAEs), combines with polyolefin plastic molecules by a form of non-covalent bonds, which can be continuously released into the environment (air, soil, water, food, etc.) and enter the organism through ingestion, inhalation or dermal exposure to produce toxic effects on health [1]. Because of its wide spread usage and high production volume, it has posed a considerable interest nowadays. Daily human exposure to DEHP in general population is estimated to be in the range from 5.8 to 19 μg/kg/day, whereas exposure through medical settings may exceed up to 167.9 mg/day [2]. Additionally, it rapidly degraded after entering the body, the US Environmental Protection Agency reported that the half-life of DEHP in human averages 12 h. DEHP and its active metabolic product mono(2-ethyl-hexyl) phthalate (MEHP) have been detected in various kinds of human tissues, including the liver, blood, placenta, amniotic fluid, and early gestation villi [3,4,5]. Recent researches showed DEHP caused damage to multiple systems in the body [6,7,8,9,10,11], especially some epidemiological and animal studies have certified the toxicity of DEHP to liver [12,13,14], the mechanisms were mainly focused on stress response, cell apoptosis, and signal pathway activation. Study found that DEHP induced normal human liver cell line (LO2 cells) apoptosis through caspases-mediated mitochondrial signaling pathway and/or death receptor pathway [15]. DEHP as PPARalpha activators played an important role in liver tumorigenesis [16]. Additionally, DEHP could also affect gap-junctional intercellular communication (GJIC), peroxisomal beta-oxidation (PBOX) activity and replicative DNA synthesis to induce hepatic tumorigenesis [17]. In this study, we focused on the effect of reactive oxygen species (ROS).

ROS plays an important role in the regulation of physiological phenomena [18]. Excessive ROS can cause the polyunsaturated fatty acids in cell membranes to undergo lipid peroxidation (LPO) (one of the indexes is MDA increased). Studies have found that DEHP caused a promotion in LPO to the liver: The activity of SOD and catalase decreased, concentration of MDA, and conjugated dienes increased [19], and it also mediated the development of non-alcoholic fatty liver by promoting LPO on high-fat diets [13]. With the further discovery of researches, found that DEHP, changed the LPO levels because of the activation of SREBP-1c and PPARα-signaling pathway and redundant ROS in HepG2 cells [20] and induced liver LOP via activating the ERK/NF-κB signaling pathway in which ROS act as the pivotal mediators of the apoptotic signaling [21]. Therefore, LOP may be one of the most important causes of hepatotoxicity induced by DEHP, in which ROS was a vital medium.

At the same time, studies also reported that ROS could induce or mediate the activation of the JNK/p38MAPK signaling pathway [22]. JNK/p38MAPK signaling pathway, important member in MAPK family, is important mechanisms involved in the regulation of gene expression, cell proliferation, and death, and plays a key role in various receptor-signaling pathways [23,24]. They also found that ROS and p38 pathways might have feedback regulation, and the activation of p38 pathway promoted ROS production [25]. Subsequent studies have shown that para-phenylenediamine induced apoptosis of hepatocytes and capsaicin result in hepatoma cell apoptosis were all via promoting the activation of p38MAPK/JNK signaling pathway and the formation of ROS [26,27]. Meanwhile, p53 is the key target of the JNK/p38MAPK signaling pathway, played a significant character in DEHP-induced hepatotoxicity [28]. Nevertheless, the role of the above mechanism in hepatotoxicity induced by DEHP has not been studied thoroughly.

This study was designed to investigate whether the activation of JNK/p38MAPK/p53 signaling pathway and further generation of ROS are involved in DEHP-induced hepatotoxicity in mice.

## 2. Materials and Methods

### 2.1. Animals and Treatment

Forty ICR mice (21 day-old), half female and half male, weighting about 19–21 gram (g), were obtained from the experimental animal center of Fujian Medical University, the animal license number is SCSK 2016-0001. The animals were quarantined for a week before the experiment. The mice were kept in standard cages and given free drinking water and food. The animal room was kept in a 12 h light-dark cycle with a temperature of 22 ± 1 °C and a relative humidity of 50 ± 5%.

Forty mice were randomly divided into four groups (*n* = 10/group, half female and half male) according to body weight and were exposed with DEHP dissolved in corn oil (Yijia, Lanzhou China) (125, 250, or 375 mg/kg/day) (Purity: 99%, Sigma-Aldrich, St. Louis, MO, USA) and corn oil (control group) by intragastrical gavage for 28 days, and the gastric capacity was 10 ml/kg. The dosage selection for exposure was based on the previous study [7]. The body weight of the mice was weighed daily and the feeding, the activity of mice was observed. Previous study has revealed that the half-life of DEHP in liver is about 24 h, then it is broken down into other metabolites [29], so all mice were sacrificed for collecting liver and blood within 24 h after the last DEHP exposure. The procedures for animal experiments were in accordance with the Guide for the Care and Use of Laboratory Animals at Fujian Medical University involving animal care (Publication No. 85-23, revised 1985), euthanasia, and tissue collection.

### 2.2. Liver Histopathological Evaluation 

After sacrificed, the liver tissues were fixed in Bouin’s solution for 1 h, after tissue dehydration and embedding, cut into a thickness of 5 μm to prepare paraffin sections. Then, the sections were stained with hematoxylin and eosin (H&E) and evaluated for histopathological changes under optical microscopy (Olympus, Tokyo, Japan).

### 2.3. Determination of Serum Biochemical Indicators

Blood was taken from the eyeball, after standing for 3 h, centrifuged at 4 °C and 3000 r/min for 15 min to prepare serum. AST and ALT in serum were measured by using a microplate reader, and the procedure was followed strictly in accordance with the kit instructions. The kits were purchased from Nanjing Jiancheng Bioengineering Research Institute.

### 2.4. Determination of Liver Oxidative Stress and LPO

The detection of ROS was followed the kit instructions (Nanjing Jiancheng Bioengineering Research Institute, Nanjing, China). Fresh liver tissue, weighting 0.5 g, homogenized with 5 mL phosphate buffer solution (PBS) by hand-held homogenizer in an ice bath to 10% liver tissue homogenate, centrifuged at 4 °C, 4000 rpm/min for 10 min to gain the supernatant as whole cell lysates. Whole cell lysates (100 μL) were incubated with 20 μM-1 mM 2,7-dichlorodihydrofluorescein diacetate (DCFH-DA) at 37 °C in the dark for 30 min to 1 h. 2,7-dichlorodihydrofluorescein (DCF) fluorescence was measured within 30 min using fluoresce microplate reader at 485 nm excitation and 525 nm emission wavelengths. In the meantime, the protein of 100 μL whole cell lysates was measured by the BCA Protein Assay Kit (Nanjing Jiancheng Bioengineering Research Institute, Nanjing, China). The result showed as fluorescence density/mg protein.

Additionally, the above whole cell lysates were measured the MDA and SOD used a microplate reader, all the procedures were followed strictly in accordance with the kit instructions (Nanjing Jiancheng Bioengineering Research Institute, Nanjing, China). The results were converted by protein quantitation.

### 2.5. RNA Extraction and Real-Time PCR Analysis

The total RNA of liver was extracted with the Trizol reagent (Invitrogen, Carlsbad, CA, USA) and its concentration and purity were determined by measuring the absorbance ratio of 260 nm/280 nm with Ultra-micro UV-visible spectrophotometer (Denovix, Wilmington, DE, USA). The cDNA was obtained by reverse transcription from 1ug RNA with PrimeScript RT reagent Kit (Takara Biotechnology, Dalian, China). The PCR procedure was carry out with SYBR Green I fluorochrome (SYBR®Premix Ex Taq™, Takara Biotechnology, Dalian, China) and LightCycler480 System (Roche, Basle, Switzerland). Additionally, they first establish an amplification system according to the kit: 10 uL SYBR Premix Ex Taq, 0.8 uL forward and reverse primer (10 μM), 2uL cDNA and RNase free dH2O to20 uL. Then, a two-step reaction procedure for PCR amplification reaction was used: The first step is 95 °C pre-denaturation for 30 seconds, the second step PCR reaction is 95 °C for 5 seconds, 60 °C for 34 seconds, one cycle, and a total of 40 cycles. The primer sequences were synthesized in Sangon Biological Technology (Shanghai, China), shown in Table 1. GAPHD was used as reference gene to calculate the relative mRNA expression of JNK, p38MAPK, and p53 by using the comparative Ct (2^−△△Ct^) method [30]. 

### 2.6. Western Blot

The total protein of liver was acquired with the RIPA lysis buffer and protease inhibitor (Beyotime Institute of Biotechnology, Shanghai, China) and its concentration was determined with the BCA Protein Assay Kit (Beyotime Institute of Biotechnology, Shanghai, China). 50 ug of protein was electrophoresed with sodium dodecyl sulfate-polyacrylamide (SDS-PAGE) gel on 5% separation and 10% spacer gel and then transferred into the poly-vinylidene fluoride (PVDF) membrane. The membrane was blocked with 5% skim milk for 2 h at room temperature and incubated at 4 °C overnight with the prime antibody: Mouse anti-JNK (1:500, sc-7345, Santa Cruz Biotechnology, Santa Cruz, CA, USA), mouse anti-phospho-JNK (1:1000, #9255, Cell Signaling Technology, Boston, MA, USA), rabbit anti-p38MAPK (1:800, #8690, Cell Signaling Technology, Boston, MA, USA), rabbit anti-phospho-p38MAPK (1:1000, #9215, Cell Signaling Technology, Boston, MA, USA), mouse anti-p53(1:500, sc-47698, Santa Cruz Biotechnology, Santa Cruz, CA, USA), and mouse anti-β-actin (1:1000, Santa Cruz Biotechnology, Santa Cruz, CA, USA). After washing with TBS containing 0.1%Tween-20, the membrane was incubated with the second antibodies conjugated to HRP: Goat anti mouse or rabbit (1:4000, BIOSS, Beijing, China) for 1 h at room temperature. The stripe was visualized with ECL plus Detection Reagent (Applygen Technologies, Beijing, China) and was analyzed with Image J. Intensity of β-actin as the standard to calculate the expression of the target protein.

### 2.7. Bisulfite Sequencing PCR

CpG islands in the promoter region of p38ɑMAPK and p53 were predicted through MethPrimer online software (http://www.urogene.org/cgi-bin/methprimer/methprimer.cgi). To prevent DNA fragmentation during bisulfite sequencing, the CpG islands of p53 gene promoter region were divided into three separate sections for amplification and detection (p53-1, p53-2, and p53-3), followed by three-part results for analysis.

The total genomic DNA was gathered from liver by using DNA extraction kit (SK8224, Sangon Biological Technology, Shanghai, China). DNA concentration and purity were determined based on the absorbance at 260 and 280 nm. The primer sequence was designed by Epidesigner (http://epidesigner.com/) [31]. The bisulfite- treated DNAs were amplified by PCR for analysis of genes promoter regions by using bisulfite sequencing specific primer pairs, as shown in Table 1.

Genomic DNA (2 ug) was treated with sodium bisulfite, amplified by PCR and using bisulfite sequencing specific primer pairs. The amplified product was recovered and purified using the kit (SK1141, Sangon Biological Technology, Shanghai, China). Following the purified product was connected to pUC18-T vector and the positive clones were filtrated by ampicillin antibiotic selection and authenticated by PCR. Finally, five positive clones were randomly selected for DNA sequencing in Sangon Biological Technology (Shanghai, China).

### 2.8. Statistical Analysis

Statistical software SPSS 23.0 (IBM, Armonk, NY, USA) was used for statistical analysis. The difference of DNA methylation level between groups was analyzed by Chi-square test and Fisher exact test. For the other data, with homogeneity of variance, Least-Significant Difference (LSD) test was used to compare the differences between groups, while Student’s *t*-test was used when the variances were different. A P-value of less than 0.05 was considered statistically significant and the results are expressed as means ± standard deviations (SD) and percentage.

## 3. Results

### 3.1. DEHP Exposure Has No Effect on Mice Body Weight

**Figure 1:**
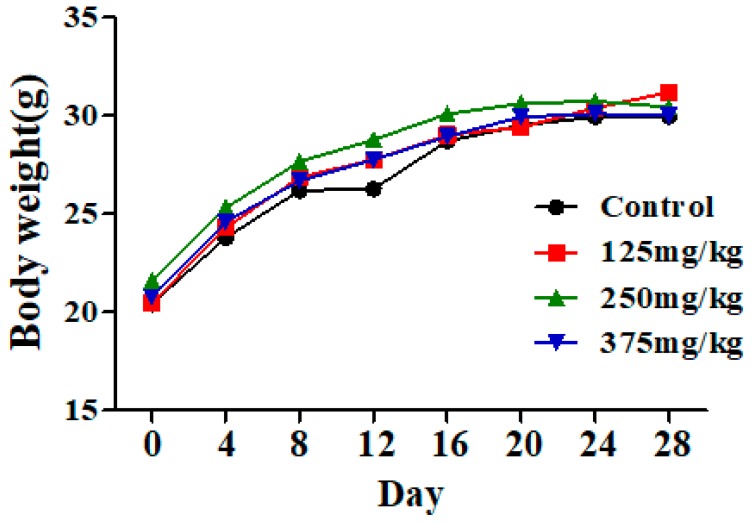
Effect of DEHP exposure on the body weight of mice (*n* = 10). The result showed that the body weight of mice in the control group and DEHP-exposed groups exhibited no significant changes. Data represented as means.

The mice had normal food consumption and activity during exposure to DEHP, and had no obvious toxicity of performance and death. There also was no significant change in body weight in the DEHP groups compared to the control group (Figure 1).

### 3.2. DEHP Induced Liver Damage and Function Disorder in Mice

**Figure 2:**
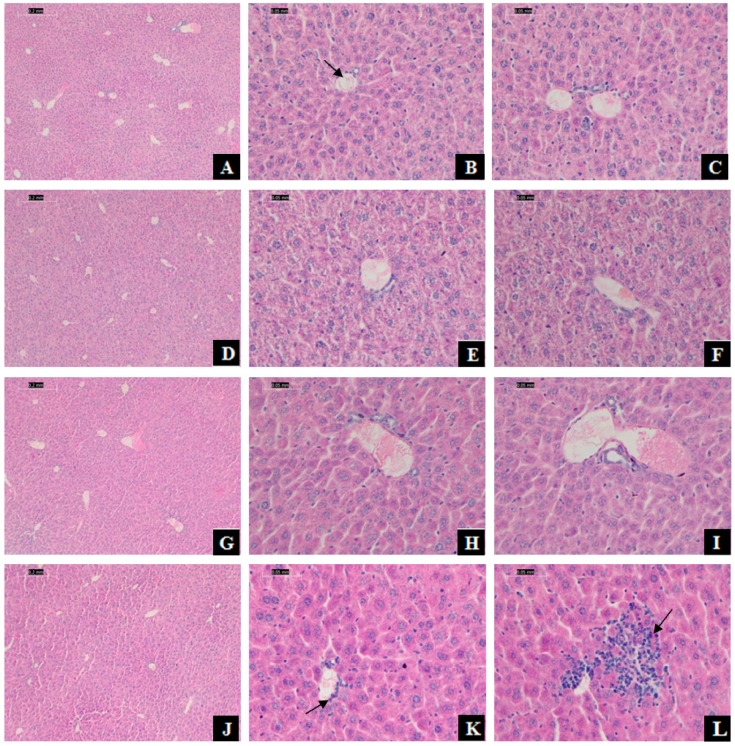
Effect of DEHP exposure on histopathology of mice liver. Photomicrographs of sections stained with hematoxylin-eosin (H&E) from mice liver tissue. Scale bar was 0.2 and 0.5 mm. (**A**–**C**): Hepatic cross-sections from the control group (treated with corn oil), with the normal hepatic lobules and cell structure, the cells radially arranged around the central vein (B: arrowhead) to form the hepatic cord; (**D**–**F**): Hepatic cross-sections from 125 mg/kg DEHP group; (**G**–**I**): Hepatic cross-sections from 250 mg/kg DEHP group; (**J**–**L**): Hepatic cross-sections from 375 mg/kg DEHP group with a small agglomeration of leukocytes in the venule (K: arrowhead) and inflammatory cells infiltration (L: arrowhead).

The hepatic histomorphology of mice has appeared injuries. After H&E staining, normal liver tissue structure and cell morphology were presented in the control group, and the liver cords were arranged orderly (Figure 2A–C). In comparison, a small agglomeration of leukocytes in the venule and inflammatory cells infiltration was observed in the 375 mg/kg DEHP-exposed group (Figure 2K,L).

**Figure 3:**
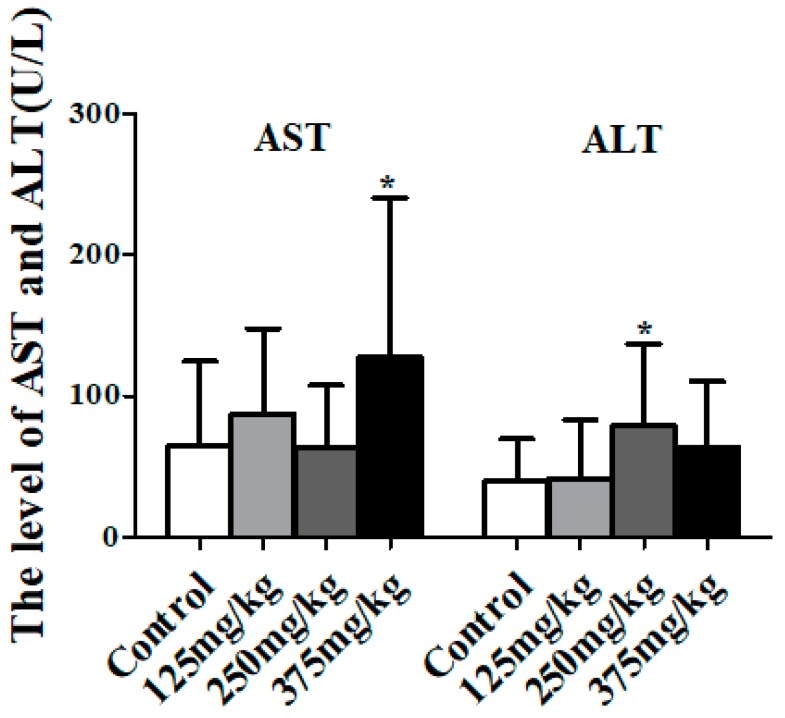
Effect of DEHP exposure on the indicators of liver damage in mice serum (*n* = 10). Data shows the activity of aspartate transaminase (AST) and alanine transaminase (ALT), represented as means ± SD, * *p* < 0.05 compared with the control group.

As Figure 3 shows, the activity of AST in mice serum of the 375 mg/kg DEHP treatment group had a significant increase compared to the control group (*p* < 0.05). Additionally, compared to the control group, the activity of ALT in mice serum significantly increased to 78.61 U/L in the 250 mg/kg DEHP treatment group (*p* < 0.05). 

### 3.3. DEHP Induced LPO in Mice Liver

**Figure 4:**
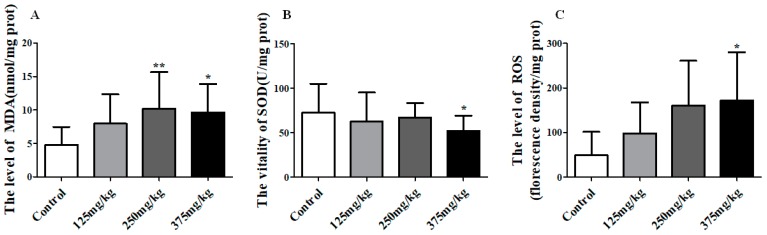
Effect of DEHP exposure on the level of oxidative stress in liver homogenate of mice (*n* = 10). (**A**) the level of MDA; (**B**) the activity of SOD; (**C**) the level of ROS. Data represented as means ± SD, * *p* < 0.05, ** *p* < 0.01 compared with the control group.

Besides, there was an obvious influence on the liver LPO (including ROS, MDA, and SOD) under the exposure of DEHP. DEHP application significantly increased the level of MDA in liver when compared to control, and reaching up to 10.19 and 9.61 nmol/mg prot in the 250 and 375 mg/kg DEHP-exposed groups, (*p* < 0.01, *p* < 0.05) (Figure 4A). Figure 4B shows the SOD activity in liver noticeably declined to 52.13 U/mg prot in the 375 mg/kg group (*p* < 0.05). In Figure 4C, there was an upward trend in the level of ROS in the liver, reaching a top of 172.20 in the 375 mg/kg DEHP group which was markedly higher than the control group (*p* < 0.05).

### 3.4. DEHP Has Effect on the Activation of JNK/p38MAPK/p53 Pathway

**Figure 5:**
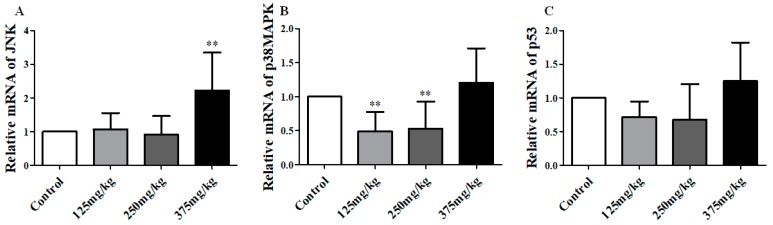
Effect of DEHP exposure on the mRNA expression of JNK/p38MAPK/p53 in liver of mice (*n* = 3). (**A**) The mRNA expression of JNK; (**B**) The mRNA expression of p38MAPK; (**C**) The mRNA expression of p53. Data represented as means ± SD, ** *p* < 0.01 compared with the control group.

We detected the mRNA and protein/phosphoprotein expression of JNK, p38MAPK and p53 in mice’s liver, and found the results as below. The mRNA analysis results are showed in Figure 5. In the 375 mg/kg DEHP-exposed group, the level of JNK mRNA was higher than the control group, about 1.2 (*p* < 0.01), and no significant changes were found in other groups (Figure 5A). On the contrary, the expression of p38 mRNA was markedly decreased in the 125 and 250 mg/kg DEHP-exposed group compared to the control group (*p* < 0.01) (Figure 5B). However, there were no significant changes in p53 mRNA.

**Figure 6:**
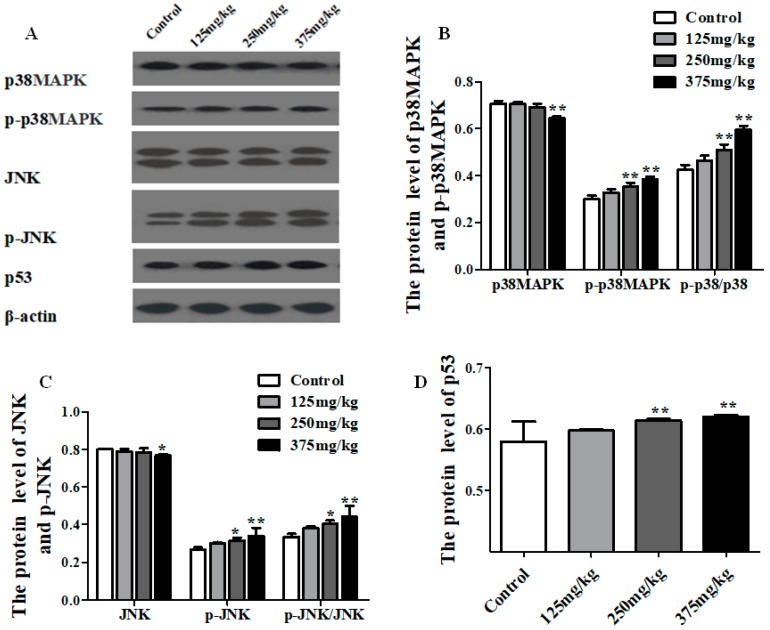
Effect of DEHP exposure on the total protein and phosphorylated protein expression of JNK/p38MAPK/p53 pathway in liver of mice (*n* = 3). (**A**) Protein bands from the experiment of Western blot; (**B**) The total protein and phosphorylated protein expression of p38MAPK; (**C**) The total protein and phosphorylated protein expression of JNK; (**D**) The total protein expression of p53. Data represented as means ± SD, * *p* < 0.05, ** *p* < 0.01 compared with the control group.

The protein expressions of the JNK, p-JNK, p38MAPK, p-p38MAPK, and p53 are shown in Figure 6. Compared with the control group, the level of p38MAPK protein showed a dramatic increase in 375 mg/kg DEHP treatment group (*p* < 0.01), and the level of p-p38MAPK protein was on the rise at the higher level of dose, especially in 250 and 375 mg/kg DEHP treatment groups, which had a statistical difference (*p* < 0.01). In addition, the ratio of p-p38MAPK to p38MAPK markedly risen in the 250 and 375 mg/kg DEHP treatment groups (*p* < 0.01) (Figure 6B), and the same trend was also found in the results of JNK and p-JNK (Figure 6C). As shown in Figure 6D, the expression of p53 protein remarkably increased, particularly in 250 and 375 mg/kg DEHP-exposed groups (*p* < 0.01).

### 3.5. DEHP Induced -Hypomethylation of the Promoter Region of p53 Gene

**Figure 7:**
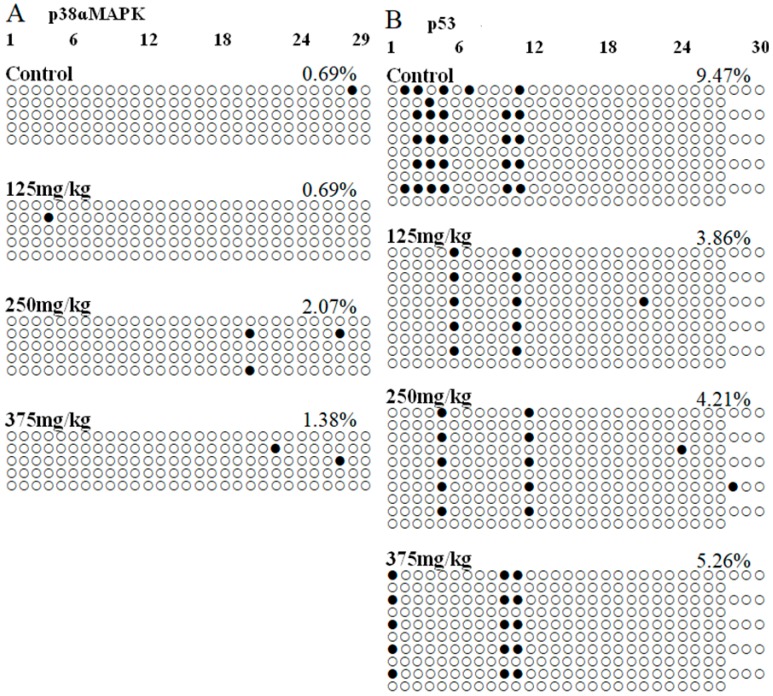
Effect of DEHP exposure on p38ɑMAPK and p53 gene promoter region methylation levels in the liver of mice (*n* = 3).

BSP experiment was performed to identify the DNA methylation level of the p38ɑMAPK and p53 promoter regions. Using the online tools to predict genes’ promoter regions that showed in Figure 7, a 289-bp fragment containing 29 CpG sites at the 5’ end of the p38ɑMAPK gene, ranging from 0 to 2000 bp fragment upstream of the transcription initiation site, was chosen for bisulfite sequencing. Moreover, a 1049-bp fragment containing 57 CpG sites at the 5’ end of the p53 gene was chosen for bisulfite sequencing. There was different level of the DNA methylation in each CpG site. Results showed in Table 2, the total methylation percentages of p38MAPK gene promoter region in the 0, 0.5, 2.0, and 8.0 mg/kg DEHP treatment group were 0.69%, 0.69%, 2.07%, and 1.38%, respectively. By statistical analysis, there was no significant difference in DEHP treatment groups compared with the control group. In addition, the total DNA methylation percentages of p53 in the control group was 9.47%, that in DEHP treatment groups was respectively 3.86%, 4.21%, and 5.26%, and the total DNA methylation percentages of p53 gene promoter region in 125 and 250 mg/kg DEHP treatment group were obviously lower to that of the control group (*p* < 0.01, *p* < 0.05).

Bisulfite sequencing and PCR-based single molecule sequencing analysis were performed. Each row represents an individually sequenced clone, and each circle represents a CpG residue. White and black circles indicate non-methylated and methylated cytosines, respectively. In each group, five positive clones were randomly collected for DNA sequencing analysis. A: p38ɑMAPK gene, B: p53 gene.

## 4. Discussion

Previous researches have established that a sequence of obvious hepatotoxicity was caused by DEHP exposure. The effect of DEHP-exposed on hepatic histopathology showed focal necrosis with inflammatory cells, fatty degeneration and the liver cytoplasm with vacuolar degeneration [32,33]. In this study, we also found a mild inflammation in liver tissue after DEHP exposure, liver histopathological results showed a small agglomeration of leukocytes in the venule and inflammatory cells infiltration exposed to 375 mg/kg/d DEHP. On the other hand, we found hepatic insufficiency. The results showed that the activity of AST and ALT in serum of mice significantly enhanced after exposure to DEHP. And AST and ALT mainly exist in liver cells, they will metastasize into serum if liver damaged. Liver tissue injury and hepatic insufficiency caused by DEHP also were reported in other studies [34]. However, the effects on ALT appear to be more profound following 250 mg/kg/d DEHP exposure than with a 375 mg/kg/d DEHP exposure. The effects of DEHP on liver function exhibit a nonmonotonic dose response. A nonmonotonic dose response is typical among common DEHP, suggesting that different effects and mechanisms may exist at different doses [7].

Then, we performed more experiments to explore the mechanism that DEHP induced liver damage and function disorder in mice and found ROS induced LPO maybe one of the reasons. ROS played an important role in the regulation of physiological phenomena [18]. Excessive ROS reacts with polyunsaturated fatty acids to form LPO products such as MDA and 4-hydroxynonenal (HNE), thereby making cell membrane fluidity changes in permeability and ultimately lead to injury in cell structure and function [35]. In our results, we found that the contents of ROS and MDA increased prominently in liver after DEHP exposure. These results provided evidence for our hypothesis that the chosen doses of DEHP induced liver injury in cell structure and function through promoting the production of ROS and activated LPO in mice liver and these results were also found in the other research [36]. Moreover, ROS are free radicals and excess ROS produce LPO result in cell lipid accumulation or cell death. Additionally, as an important active substance in the body, SOD can effectively remove free radicals and alleviate damage caused by peroxidation. In this study, the activity of SOD significantly decreased, which further strengthens the negative effect of ROS in liver after DEHP exposure. More interesting, we found the marked increase of ROS in mice liver had a positive relationship with the DEHP dose, correspondingly, SOD decreased with DEHP dose. Thus it is worth to speculate LPO was one of the most important causes of hepatotoxicity induced by DEHP, while ROS may be a vital medium in it. Ghosh et al [21] have put forward DEHP induced hepatocyte apoptosis through ROS and Zhang et al [20] found that the production of excessive ROS as a result of the treatment of DEHP had a distinct effect on the MDA and SOD and thereby aroused cell lipid accumulation even death. The results of these studies were similar to our results, which clearly verified our speculations.

In addition, we further assessed how DEHP altered ROS in mice liver. Excessive ROS generation could be induced by a series of causes, in which the effect of certain signaling pathways activation is non-ignorable. JNK/p38MAPK/p53 signaling pathways activation is closely related to the production of excessive ROS. Draw on the previous researches, we have known that JNK/p38MAPK signaling pathways might be critical mediators of chlorpyrifos-induced neuronal cell apoptosis by generating ROS [37] and JNK/p38MAPK signaling pathways inhibitor decreased the generation of ROS to reduce HepaRG cell death [38]. There may be positive feedback regulation between JNK/p38MAPK signaling pathway and ROS. Meanwhile a review adequately elaborated that p53 can mediate ROS production through a variety of pathways, including JNK/p38MAPK/p53 signaling pathways activation [39]. In this study, the results showed that DEHP has an effect on the activation of JNK/p38MAPK/p53 signaling pathways. To be more specific, the expression of p-JNK and p-p38MAPK protein was apparently increased with the effect of DEHP and the proportion of them in respective total protein also has a significant augment. Moreover, result of observable rise in the level of p53 protein was detected in mice liver, which was consistent with a previous study [28]. In this situation, DEHP could induce JNK and p38 phosphorylation to activate the JNK/p38MAPK/p53 signaling pathways and then increased p53 expression. Thus, we have drawn an inference that after short-term exposure to DEHP, JNK/p38MAPK/p53 signaling pathways was activated in the liver of mice, which could mediate the production of superfluous ROS. 

Additionally, to find more about DEHP’s effect on this signaling pathway, epigenetics reason also cannot be ignored [40,41,42]. Our results showed that DEHP altered JNK, p38MAPK and p53 genes mRNA has no linear with protein. This also found in our previous study [10]. For some special genes, the relation between mRNA and protein is not strictly linear, different regulation mechanisms acting on both the synthesized mRNA and the synthesized protein. Then we used epigenetic experiment to further assess how DEHP altered mRNA expression for the encoding genes of these proteins. To our knowledge, there were few vivo experiments on DEHP induction of DNA methylation modification in liver, especially in JNK/p38MAPK/p53 signaling pathway. In this study, we speculate that DEHP has negative effects on the liver through changing the DNA methylation level of these signaling pathways’ two important genes (p38MAPK and p53). However, we only have observed that DNA methylation level of the p53 gene promoter region in mouse liver was significantly down-regulated after DEHP exposure, which was consistent with the expression of in p53 mRNA. The result manifested DEHP altered the expression of the p53 gene through affecting the DNA methylation level in the promoter region of p53, which maybe cause the change of JNK/p38MAPK/p53 signaling pathways. Epigenetic is an important mechanism for the capability of environmental chemistry to affect health and disease. Phenotypic changes due to environmental exposure are regulated by epigenetic gene programs in various tissues [43]. DEHP can change the p53 gene’s DNA methylation level and induce hypomethylation and caused more damage in mice tissue, which further confirm the epigenetics as an important mechanism of environmental chemicals on the body.

## 5. Conclusions

In conclusion, short-term exposure to DEHP has toxic effect in mice liver. The possible molecular mechanism is that the DNA methylation level in the promoter region of p53 gene is affected after DEHP exposure, which may activate the JNK/p38MAPK/p53 signaling pathway and cause the excess production of ROS. ROS may be a vital target for DEHP-induced hepatotoxicity in mice. However, the specific mechanism remains to be further verified in vitro cell experiments.

## Figures and Tables

**Table 1:** Gene primer sequences for amplification.

Target Gene	Primer Sequences
Real-time PCR	
GAPDH(141dp)	Forward: 5′-GCAAGTTCAACGGCACAG-3′
	Reverse: 5′ -CGCCAGTAGACTCCACGAC-3′
JNK(128dp)	Forward: 5′-ATGCAAATCTTTGCCAAGTG-3′
	Reverse: 5′-AGGCTTTAAGTCCCGATGAA-3′
p38 MAPK(195dp)	Forward: 5′-AAGCCATGAGGCAAGAAACT-3′
	Reverse: 5′-TCATCAGGGTCGTGGTACTG-3′
p53(195dp)	Forward: 5′-AAGCCATGAGGCAAGAAACT-3′
	Reverse: 5′-TCATCAGGGTCGTGGTACTG-3′
Bisulfite sequencing PCR	
p38ɑ MAPK	Forward: 5′-CTGGCTCTGCAGGTGACCCAAGCAG-3′
	Reverse: 5′ -AGGAGAAGGGGTCCCTGCT-3′
p53-1	Forward: 5′-TTAAGGGGAAACTCCTGAAGATG-3′
	Reverse: 5′-GGAAGACTCGCATGTTCAGAAACATC-3′
p53-2	Forward: 5′-GCTAGCTGGGGTTGGTCATCACCAC-3′
	Reverse: 5′-AGTGTCCAAAGCCAAGCGCCTAGG-3′
p53-3	Forward: 5′-AAGCCGAACCTAAAGCAATCACCAGGG-3′
	Reverse: 5′-GCCTGCAGAGGGCGCATAATTTCTA-3′

**Table 2:** Effect of DEHP exposure on p38ɑMAPK and p53 gene promoter region methylation levels in the liver of mice (*n* = 3).

Gene	Group	Methylated CpG Sites Numbers	Non-Methylated CpG Sites Numbers	The Total Methylation Percentages
p38ɑMAPK	Control	1	144	0.69%
	125 mg/kg	1	144	0.69%
	250 mg/kg	3	142	2.07%
	375 mg/kg	2	143	1.38%
p53	Control	27	258	9.47%
	125 mg/kg	11	274	3.86% **
	250 mg/kg	12	273	4.21% *
	375 mg/kg	15	270	5.26%

To confirm the CpG islands methylation levels of the p38ɑMAPK and p53 promoter regions, bisulfite sequencing was utilized to determine the total methylation percentages at the whole CpG islands within the p38ɑMAPK and p53 promoter regions. Data represented as percentages, * *p* < 0.05, ** *p* < 0.01 compared with the control group.

## References

[1] Erythropel H.C., Maric M., Nicell J.A., Leask R.L., Yargeau V. (2014). Leaching of the plasticizer di(2-ethylhexyl)phthalate (DEHP) from plastic containers and the question of human exposure. Appl. Microbiol. Biotechnol..

[2] Kavlock R., Boeckelheide K., Chapin R., Cunningham M., Faustman E., Foster P., Golub M., Henderson R., Hinberg I., Little R. (2002). NTP center for the evaluation of risks to human reproduction: Phthalates expert panel report on the reproductive and developmental toxicity of di-n-octyl phthalate. Reprod. Toxicol..

[3] Matsumoto M., Hirata-Koizumi M., Ema M. (2008). Potential adverse effects of phthalic acid esters on human health: A review of recent studies on reproduction. Regul. Toxicol. Pharmacol..

[4] Shiota K., Nishimura H. (1982). Teratogenicity of di(2-ethylhexyl) phthalate (DEHP) and di-n-butyl phthalate (DBP) in mice. Environ. Health Perspect..

[5] Heudorf U., Mersch-Sundermann V., Angerer J. (2007). Phthalates: Toxicology and exposure. Int. J. Hyg. Environ. Health.

[6] Hannon P.R., Brannick K.E., Wang W., Flaws J.A. (2015). Mono(2-ethylhexyl) phthalate accelerates early folliculogenesis and inhibits steroidogenesis in cultured mouse whole ovaries and antral follicles. Biol. Reprod..

[7] Liu J., Wang W., Zhu J., Li Y., Luo L., Huang Y., Zhang W. (2018). Di(2-ethylhexyl) phthalate (DEHP) influences follicular development in mice between the weaning period and maturity by interfering with ovarian development factors and microRNAs. Environ. Toxicol..

[8] Ha M., Guan X., Wei L., Li P., Yang M., Liu C. (2016). Di-(2-ethylhexyl) phthalate inhibits testosterone level through disturbed hypothalamic-pituitary-testis axis and ERK-mediated 5alpha-Reductase 2. Sci. Total Environ..

[9] Pei X., Duan Z., Ma M., Zhang Y., Guo L. (2014). Role of Ca/CaN/NFAT signaling in IL-4 expression by splenic lymphocytes exposed to phthalate (2-ethylhexyl) ester in spleen lymphocytes. Mol. Biol. Rep..

[10] Zarean M., Keikha M., Poursafa P., Khalighinejad P., Amin M., Kelishadi R. (2016). A systematic review on the adverse health effects of di-2-ethylhexyl phthalate. Environ. Sci. Pollut. Res. Int..

[11] Wu Y., Li K., Zuo H., Yuan Y., Sun Y., Yang X. (2015). Primary neuronal-astrocytic co-culture platform for neurotoxicity assessment of di-(2-ethylhexyl) phthalate. J. Environ. Sci..

[12] Wang W., Xu X., Fan C. (2015). Health hazard assessment of occupationally di-(2-ethylhexyl)-phthalate-exposed workers in China. Chemosphere.

[13] Chen H., Zhang W., Rui B.B., Yang S.M., Xu W.P., Wei W. (2016). Di(2-ethylhexyl) phthalate exacerbates non-alcoholic fatty liver in rats and its potential mechanisms. Environ. Toxicol. Pharmacol..

[14] Maranghi F., Lorenzetti S., Tassinari R., Moracci G., Tassinari V., Marcoccia D., Di Virgilio A., Eusepi A., Romeo A., Magrelli A. (2010). In utero exposure to di-(2-ethylhexyl) phthalate affects liver morphology and metabolism in post-natal CD-1 mice. Reprod. Toxicol..

[15] Yang G., Zhang W., Qin Q., Wang J., Zheng H., Xiong W., Yuan J. (2015). Mono(2-ethylhexyl) phthalate induces apoptosis in p53-silenced L02 cells via activation of both mitochondrial and death receptor pathways. Environ. Toxicol..

[16] Ren H., Aleksunes L.M., Wood C., Vallanat B., George M.H., Klaassen C.D., Corton J.C. (2010). Characterization of peroxisome proliferator-activated receptor alpha-independent effects of PPARalpha activators in the rodent liver: di-(2-ethylhexyl) phthalate also activates the constitutive-activated receptor. Toxicol. Sci..

[17] Isenberg J.S., Kamendulis L.M., Smith J.H., Ackley D.C., Pugh G., Lington A.W., Klaunig J.E. (2000). Effects of di-2-ethylhexyl phthalate (DEHP) on gap-junctional intercellular communication (GJIC), DNA synthesis, and peroxisomal beta oxidation (PBOX) in rat, mouse, and hamster liver. Toxicol. Sci..

[18] Ray P.D., Huang B.W., Tsuji Y. (2012). Reactive oxygen species (ROS) homeostasis and redox regulation in cellular signaling. Cell. Signal..

[19] Santhosh A., Nair K.G., Arun P., Deepadevi K.V., Manojkumar V., Lakshmi L.R., Kurup P.A. (1998). Effect of DEHP [di-(2-ethyl hexyl) phthalate] on lipid peroxidation in liver in rats and in primary cultures of rat hepatocytes. Indian J. Med. Res..

[20] Zhang W., Shen X.Y., Zhang W.W., Chen H., Xu W.P., Wei W. (2017). The effects of di 2-ethyl hexyl phthalate (DEHP) on cellular lipid accumulation in HepG2 cells and its potential mechanisms in the molecular level. Toxicol. Mech. Methods..

[21] Ghosh J., Das J., Manna P., Sil P.C. (2010). Hepatotoxicity of di-(2-ethylhexyl)phthalate is attributed to calcium aggravation, ROS-mediated mitochondrial depolarization, and ERK/NF-kappaB pathway activation. Free Radic. Biol. Med..

[22] Son Y., Cheong Y.K., Kim N.H., Chung H.T., Kang D.G., Pae H.O. (2011). Mitogen-activated protein kinases and reactive oxygen species: How can ROS activate MAPK pathways?. J. Signal. Transduct..

[23] Huang G., Tang B., Tang K., Dong X., Deng J., Liao L., Liao Z., Yang H., He S. (2014). Isoquercitrin inhibits the progression of liver cancer in vivo and in vitro via the MAPK signalling pathway. Oncol. Rep..

[24] Zhang B., Wu T., Wang Z., Zhang Y., Wang J., Yang B., Zhao Y., Rao Z., Gao J. (2015). p38MAPK activation mediates tumor necrosis factor-alpha-induced apoptosis in glioma cells. Mol. Med. Rep..

[25] Duan W. (2010). Mechanism Research of Natural Drug Silybin and Evodiamine Induced Death of Human Fibrosarcoma HT1080 Cells.

[26] Chye S.M., Tiong Y.L., Yip W.K., Koh R.Y., Len Y.W., Seow H.F., Ng K.Y., Ranjit de A., Chen S.C. (2014). Apoptosis induced by para-phenylenediamine involves formation of ROS and activation of p38 and JNK in chang liver cells. Environ. Toxicol..

[27] Bu H.Q., Cai K., Shen F., Bao X.D., Xu Y., Yu F., Pan H.Q., Chen C.H., Du Z.J., Cui J.H. (2015). Induction of apoptosis by capsaicin in hepatocellular cancer cell line SMMC-7721 is mediated through ROS generation and activation of JNK and p38 MAPK pathways. Neoplasma.

[28] Ha M., Wei L., Guan X., Li L., Liu C. (2016). p53-dependent apoptosis contributes to di-(2-ethylhexyl) phthalate-induced hepatotoxicity. Environ. Pollut..

[29] Mittermeier A., Volkel W., Fromme H. (2016). Kinetics of the phthalate metabolites mono-2-ethylhexyl phthalate (MEHP) and mono-n-butyl phthalate (MnBP) in male subjects after a single oral dose. Toxicol. Lett..

[30] Livak K.J., Schmittgen T.D. (2001). Analysis of relative gene expression data using real-time quantitative PCR and the 2(-Delta Delta C(T)) Method. Methods.

[31] Weng S., Wang W., Li Y., Li H., Lu X., Xiao S., Wu T., Xie M., Zhang W. (2014). Continuous cadmium exposure from weaning to maturity induces downregulation of ovarian follicle development-related SCF/c-kit gene expression and the corresponding changes of DNA methylation/microRNA pattern. Toxicol. Lett..

[32] Ito Y., Nakamura T., Yanagiba Y., Ramdhan D.H., Yamagishi N., Naito H., Kamijima M., Gonzalez F.J., Nakajima T. (2012). Plasticizers may activate human hepatic peroxisome proliferator-activated receptor alpha less than that of a mouse but may activate constitutive androstane receptor in liver. PPAR Res..

[33] Dong X., Dong J., Zhao Y., Guo J., Wang Z., Liu M., Zhang Y., Na X. (2017). Effects of long-term in vivo exposure to di-2-ethylhexylphthalate on thyroid hormones and the TSH/TSHR signaling pathways in wistar rats. Int. J. Environ. Res. Public Health.

[34] Erkekoglu P., Zeybek N.D., Giray B.K., Rachidi W., Kizilgun M., Hininger-Favier I., Favier A., Asan E., Hincal F. (2014). The effects of di(2-ethylhexyl)phthalate on rat liver in relation to selenium status. Int. J. Exp. Pathol..

[35] Gasparovic A.C., Jaganjac M., Mihaljevic B., Sunjic S.B., Zarkovic N. (2013). Assays for the measurement of lipid peroxidation. Method. Mol. Biol..

[36] Zhang W., Shen X.Y., Zhang W.W., Chen H., Xu W.P., Wei W. (2017). Di-(2-ethylhexyl) phthalate could disrupt the insulin signaling pathway in liver of SD rats and L02 cells via PPARgamma. Toxicol. Appl. Pharmacol..

[37] Ki Y.W., Park J.H., Lee J.E., Shin I.C., Koh H.C. (2013). JNK and p38 MAPK regulate oxidative stress and the inflammatory response in chlorpyrifos-induced apoptosis. Toxicol. Lett..

[38] Wang S., Wang M., Wang M., Tian Y., Sun X., Sun G., Sun X. (2018). Bavachinin induces oxidative damage in HepaRG cells through p38/JNK MAPK pathways. Toxins.

[39] Liu B., Chen Y., St Clair D.K. (2008). ROS and p53: A versatile partnership. Free Radic. Biol. Med..

[40] Prados J., Stenz L., Somm E., Stouder C., Dayer A., Paoloni-Giacobino A. (2015). Prenatal exposure to DEHP affects spermatogenesis and sperm DNA methylation in a strain-dependent manner. PLoS ONE.

[41] Li L., Zhang T., Qin X.S., Ge W., Ma H.G., Sun L.L., Hou Z.M., Chen H., Chen P., Qin G.Q. (2014). Exposure to diethylhexyl phthalate (DEHP) results in a heritable modification of imprint genes DNA methylation in mouse oocytes. Mol. Biol. Rep..

[42] Lyu Z.Q., Xie X., Ke Y.B. (2016). Effects of long term and low dose di-(2-ethylhexyl) phthalate exposure on global genome DNA methylation in HePG2 cells. Zhonghua Lao Dong Wei Sheng Zhi Ye Bing Za Zhi.

[43] Singh S., Li S.S. (2012). Epigenetic effects of environmental chemicals bisphenol A and phthalates. Int. J. Mol. Sci..

